# First reported nosocomial SARS-CoV-2 outbreak in a hospital-based laundry facility

**DOI:** 10.1017/S0950268821002016

**Published:** 2021-09-15

**Authors:** Miki Goldenfeld, Neta Zuckerman, Sharon Amit, Ilana Tal, Sabrina Hasson, Shiraz Gefen-Halevi, Asaf Biber, Orna Mor, Gili Regev-Yochay

**Affiliations:** 1 Infection Prevention & Control Unit, Sheba Medical Center, Ramat Gan, Israel; 2 Sackler School of Medicine, Tel Aviv University, Tel Aviv, Israel; 3 Central Virology Laboratory, Ministry of Health, Tel Hashomer, Ramat Gan, Israel; 4 Clinical Microbiology, Sheba Medical Center, Ramat Gan, Israel

**Keywords:** COVID-19, epidemiological investigation, health care workers, laundromat facility, next-generation sequencing, SARS-CoV-2

## Abstract

Nosocomial severe acute respiratory syndrome coronavirus 2 (SARS-CoV-2) outbreaks among health care workers have been scarcely reported so far. This report presents the results of an epidemiologic and molecular investigation of a SARS-CoV-2 outbreak among laundromat facility workers in a large tertiary centre in Israel. Following the first three reported cases of SARS-CoV-2 among laundromat workers, all 49 laundromat personnel were screened by qRT-PCR tests using naso- and oropharingeal swabs. Epidemiologic investigations included questionnaires, interviews and observations of the laundromat facility. Eleven viral RNA samples were then sequenced, and a phylogenetic analysis was performed using MEGAX.

The integrated investigation defined three genetic clusters and helped identify the index cases and the assumed routes of transmission. It was then deduced that shared commute and public showers played a role in SARS-CoV-2 transmission in this outbreak, in addition to improper PPE use and social gatherings (such as social eating and drinking). In this study, we present an integrated epidemiologic and molecular investigation may help detect the routes of SARS-CoV-2 transmission, emphasising such routes that are less frequently discussed. Our work reinforces the notion that person-to-person transmission is more likely to cause infections than environmental contamination (e.g. from handling dirty laundry).

## Introduction

In December 2019, severe acute respiratory syndrome coronavirus 2 (SARS-CoV-2) emerged in Wuhan City, Hubei Province, China and rapidly spread worldwide, causing the coronavirus disease 2019 (COVID-19) pandemic [1]. The major route SARS-CoV-2 transmission is considered to be person-to-person transmission via droplets [2], although airborne transmission has been reported as well [3]. The role of environmental transmission, via fomites on surfaces has been largely debated [4–6], with no certain conclusion on the matter.

Initial data estimated that 3300 health care workers (HCW) were infected in China [7], and later data that emerged suggested that up to 20% of HCW in Italy have been infected by late march 2020 [7]. However, only a few epidemiologic investigations of transmission chains among HCW have been reported so far [8, 9].

Here, we report an epidemiological investigation of a COVID-19 outbreak among laundry workers in a large tertiary medical centre, that included a genomic investigation of SARS-CoV-2 strains to determine the potential routes of transmission.

## Methods

### Setting and study period

The Sheba Medical Center (SMC) is the largest tertiary-care medical centre in Israel, affiliated with Tel-Aviv University. Of its 1600 acute care beds, up to 250 have been dedicated to nine different COVID-19 admission units and departments during the three surges of the SARS-CoV-2 pandemic in 2020. Over 9500 HCW are employed at SMC, of whom over 4500 are non-clinical staff. All laundry at SMC is delivered to a single central facility. Laundry from the dedicated COVID-19 units is transported in biohazard bags, which are then loaded into biohazard containers. On arrival, the laundry is sorted at a sorting station, which is a 5 m long table. The laundry is then loaded into the laundry machines for thorough sanitation. The SMC laundromat facility employs 49 workers, of whom 12 handle the dirty laundry (sorting or loading into laundry machines). Personal protective equipment (PPE) for these workers included gloves and gowns in the pre-COVID-19 era, while N-95 masks and face shields became mandatory in March 2020. Notably, the laundromat site is an 80 m^2^ hangar with large ventilators hanging from the ceiling. From July 20 until 4 August 2020, coinciding with the second surge of COVID-19 in Israel, a SARS-CoV-2 outbreak occurred among the laundromat facility workers.

### Epidemiological investigation

Starting April 2020, every positive SARS-CoV-2 PCR case among SMC HCW initiated an epidemiological investigation by the Infection Prevention and Control Unit (IPCU). The investigations include highly detailed interviews of the positive patients and their in-hospital contacts. Those interviews addressed general information regarding household, community and work-related contacts, including the nature and duration of the contact; potential exposures to SARS-CoV-2 infected or exposed individuals (secondary exposures); COVID-19 symptoms of cases and their contacts, attendance of any high-risk sites such as gyms, religious or social gatherings; and details on PPE use. Exposed HCW were then requested to undergo home quarantine and two nasopharyngeal swabs for SARS-CoV-2 PCR if the exposure to the case was deemed significant. In cases in which the exposure was thought to be less significant, the exposed HCW were asked to continue working but undergo at least 2 SARS-CoV-2 PCR tests to rule out an infection.

In some cases, to verify the findings interviews were repeated and patients' answers cross-referenced.

An outbreak was defined following the detection of three or more cases in a single department. Outbreak investigation included further IPCU observations of the facility and ward in which it occurred, screening all HCW in that department and interviews with the management staff of the department.

### Screening

**Fig. 1.**
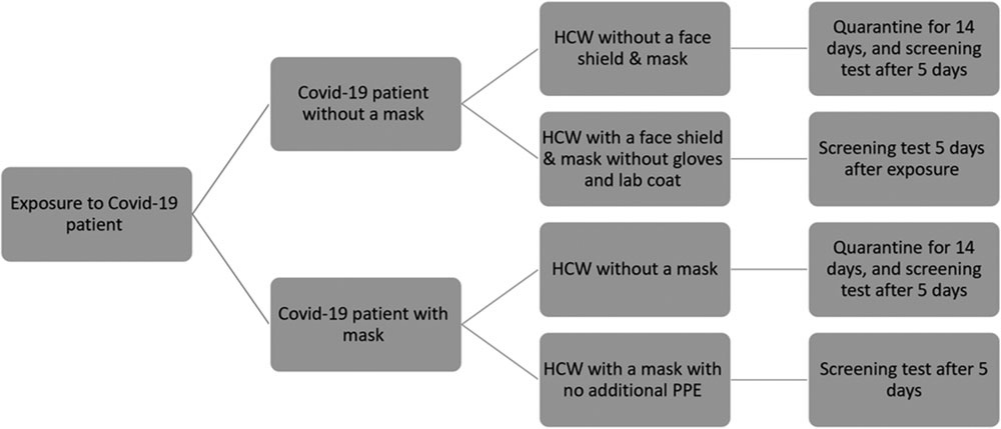
Flow chart of IPCU policy for exposure to COVID-19 positive patients.

Screening for SARS-CoV-2 by qRT-PCR was performed using naso- and oro-pharingeal swabs in the following scenarios: a. Any HCW who reported one or more of the following symptoms: fever, cough, headache, myalgia, sore throat, rhinorrhoea, unexplained severe fatigue, anosmia and ageusia. b. Any HCW who reported exposure to a suspected or detected SARS-CoV-2 positive person (Fig. 1). The screening was performed using the Allplex™ SARS-CoV-2 assay qRT-PCR following nucleic-acid extraction (Seegene Inc., S. Korea) according to the manufacturer's instructions.

### Next-generation sequencing

Extracted RNA was reverse-transcribed using SuperScriptIV (ThermoFisher Scientific, Waltham, MA, USA), and specific primers (V3, https://artic.network/ncov-2019) were used to amplify SARS-CoV-2 complete genomes with Q5 Hot-Start Polymerase (NEB). Libraries were prepared using NexteraXT (Illumina, San Diego, CA, USA), purified with AMPure XP magnetic beads (Beckman Coulter, Brea, CA, USA) and evaluated by Qubit dsDNA-HS (Thermo Fisher Scientific, Waltham, MA, USA) and TapeStation 4200 via DNA-HS D1000 (Agilent, Santa Clara, CA, USA). Libraries were sequenced on MiSeq using V3 2X300 (Illumina).

### Bioinformatics and phylogenetic analyses

Fastq files underwent processing, including quality control filtering, mapped to the reference genome (NC_045512.2), multiple alignments, construction of consensus sequence and mutation analyses via R and Bioconductor as previously described [10].

Molecular Phylogenetic analysis was applied via MEGAX [11], and the evolutionary history was inferred by using the maximum likelihood method based on the general time-reversible model with a proportion of invariable sites and gamma plus invariant site-distributed rate heterogeneity (GTR + G + I), chosen via jModelTest 2 [12]. The percentage of phylogenetic trees in which the associated taxa clustered together was determined via a bootstrap of 1000 runs.

## Results

### Outbreak description

Over the duration of 2 weeks, 14 individuals contracted SARS-CoV-2, including 11 laundromat facility workers (seven sorters, two drivers and two general workers) and three secondary cases of family members (of whom two are SMC workers as well). One was detected at the pre-symptomatic stage, seven were detected after becoming symptomatic and six remained asymptomatic throughout the period of their PCR-proven infection. The demographic details of this cohort are presented in Table 1. A total of 49 HCW were screened, and 98 PCR tests were performed during the outbreak.

**Table 1.** Demographic information of the cohorts patients and their main suspected contacts

Patient	Gender	Age	Work Station	Date of first positive PCR	Onset of symptoms	Reported patients who came in contact	Types of Reported Contacts
1	M	46	Laundromat facility, driver	22.7	21.7	–	–
2	M	37	Laundry sorting	28.7	28.7	2.a	Shared household
2.a	M	57	Dispatch centre	29.7	A-symptomatic	2	Shared household
3	M	43	Laundry sorting	29.7	28.7	7	Eating
						4	Shared commute
						8	Public showers
3.a	F	10	---	31.7	31.7	3	Shared household
4	M	58	Laundry sorting	30.7	A-symptomatic	3,6	Shared commute
5	M	52	Laundry sorting	2.8	28.7	8	Public showers
6	M	44	Laundry sorting	2.8	A-symptomatic	4	Shared commute
7	M	46	Laundry sorting	2.8	3.8	3	Eating
						7.a	Shared household
7.a	F	38	Cleaning crew	4.8	A-symptomatic	7	Shared Household
8	M	43	Laundromat facility, multiple stations	3.8	3.8	9	Social meeting
						3, 5	Public showers
9	M	42	Laundromat facility, DRiver	3.8	3.8	8	Social meeting
						10	Public showers
10	M	38	Laundromat facility, multiple stations	4.8	A-symptomatic	9	Public showers
11	M	35	Laundry sorting	4.8	3.8	–	–

**Fig. 2.**
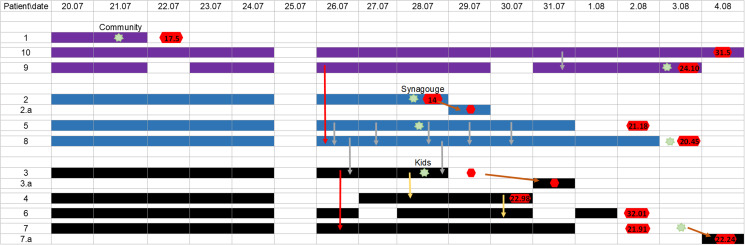
A timeline of all positive SARS-CoV-2 cases at the laundromat facility. Filled cells represent presence at work, black denotes cluster 1, as determined by NGS, blue denotes cluster 2, purple denotes cluster 3. Green star represents the first day of symptoms onset. Red hexagon represents positive RT-PCR test for SARS-CoV-2, N-gene Ct values mentioned when available. Arrows indicate contacts without proper PPE use, Red arrows represent social meetings such as eating together, yellow arrows represent shared commute, grey arrows represent shared showers and brown arrows represent contacts in between family members who live at the same household.

The first case, Patient 1, is a driver who delivers the dirty laundry to the laundromat facility. On 22 July 2020, he presented with COVID-19 symptoms and tested positive for SARS-CoV-2 infection. His suspected source of infection was a community contact, with suspected exposures during grocery shopping in a large supermarket, a week prior to his diagnosis. He did not attend any religious ceremonies or family gatherings. He did not report exposure to any known or suspected SARS-CoV-2 infected personnel at work, nor did he report intense handling of the dirty laundry or improper PPE use. A week later Patient 2, who works at the laundry sorting station, was tested due to myalgia and severe fatigue and found positive for SARS-CoV-2. His father, patient 2.a, who works at another site at SMC, also tested positive a day after his son's diagnosis, while completely asymptomatic. A day later, patient 3, who also works at the laundry sorting station, tested positive. He was tested due to a cough that he began suffering from a day earlier. Two of his children were tested within 24 h of his diagnosis and were found positive. An outbreak was then announced, since three cases were detected in a single department, and screening for SARS-CoV-2 was ordered for all personnel who attended work during that period, regardless of the epidemiological investigation's findings. Within 2 weeks, all the 49 laundromat facility workers were tested for SARS-CoV-2, apart from two workers who did not attend work during that month, due to unrelated medical conditions. In total, 11 laundromat workers were found to be positive for SARS-CoV-2 by RT-PCR test. Two other SMC personnel, patients 2.a and 7.a, are family members of patients 2 & 7. The sequence of detection of the following cases, their suspected source of infection, symptoms and demographic details are described in Figure 2 and Table 1.

### Sequencing and phylogenetic tree construction and analysis

**Fig. 3.**
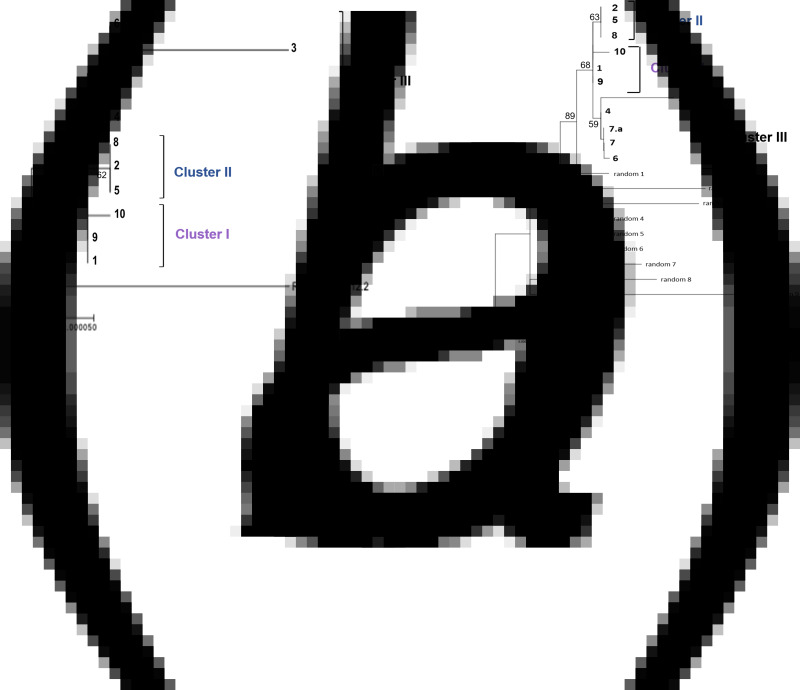
Phylogenetic tree representing laundromat outbreak. Molecular Phylogenetic analysis by maximum likelihood method, applied via MEGA7 software. The evolutionary history was inferred by using the maximum likelihood method based on the GTR + I + G model. The tree with the highest log-likelihood is shown. The percentage of trees in which the associated taxa clustered together is shown next to the branches, with a bootstrap of 1000 runs. The tree is drawn to scale, with branch lengths measured in the number of substitutions per site. The analysis involved 12 nucleotide sequences including the reference sequence NC_045512.2. (a) The phylogenetic tree of the viral samples from the 11 SMC workers. (b) An extended phylogenetic tree representing the 11 SMC workers together with 9 randomly chosen samples of patients from the general population at the same time period of the B.1.362 lineage.

Out of the 14 cases, samples from 11 patients were available for viral RNA extraction. Ten of them were laundromat workers and one is the spouse of patient 7 which is also an SMC personnel. Most samples sequenced (8/11) had >97% coverage of the SARS-CoV-2 genome, 1/11 had 81% coverage and 2/11 had 23% coverage. All 11 patients were associated with the Pangolin lineage B.1.362. A phylogenetic tree was constructed and three different clusters were detected (Fig. 3a). To verify our results an extended phylogenetic tree of the 11 patients in addition to nine randomly chosen samples of patients from the general population at the same time period of the B.1.362 lineage was constructed (Fig. 3b). The extended phylogenetic tree demonstrates the same clustering patterns. In cluster 3, which included five patients, four samples were identical, and the sample of patient 3 differed in eight nucleotides from the others. The sample of patient 3 was obtained 2 weeks after his diagnosis with SARS-CoV-2. Previous works demonstrated the development of quasi-species within 24 h [13], thus explaining the variance of patient 3 from the rest of his cluster members.

### Outbreak investigation

The first cluster, as defined by NGS, consisted of three patients (patient 1, the suspected index case, patient 9 and patient 10). While they did not recall any particular social or direct contact, they did work the same shifts during the 2 days prior to the onset of symptoms of patient 1. Patient 9 and 10 also reported concomitantly using the communal showers at the end of one of their shifts. The second cluster consisted of four patients: patients 2 and 2.a, who are father and son and patients 5 and 8. The investigation suggested that either patient 2 or 2.a are the index case of this cluster, as they attended an outdoor religious gathering in a SARS-CoV-2 endemic city. In addition, the N-gene Ct value of patient 2 was low, and indicating that the test was obtained early on in his illness. The secondary cases were patients 5 and 8, who worked the same shifts at the same station with patient 2 3 days before his diagnosis, including the day of his symptoms' onset. Additionally, they reported using the laundromat's facility communal showers at least five times. Both patient 5 and 8 did not report any potential sources of exposure in the community. The third cluster consisted of six patients: patients 3 & 3.a, patients 4, 6, 7 and 7.a. The epidemiologic investigation suggests that patient 3 is the index case of this cluster. He was suspected to have contracted the virus from his 3-year-old boy, who was completely asymptomatic but tested positive a day after his father was detected. Two secondary cases among the laundry sorting workers occurred due to exposure to patient 3 (patient 4 who commuted with patient 3 and patient 7 who dined with him), an additional secondary case was his 10-year-old daughter, patient 3.a, who was diagnosed 2 days later. Two tertiary cases were Patient 6 who commuted with patient 4 and patient 7.a who is the spouse of patient 7.

Thus, the epidemiological investigation suggested four main risk behaviours for infection transmission: (1) Not using proper PPE. (2) Social meetings which included drinking or eating. (3) Commuting together to work in private vehicles. (4) Simultaneous use of the public showers.

### Outbreak management

Following the third case of SARS-CoV-2, an outbreak in the laundromat facility was declared. To contain the outbreak, we screened all laundromat workers for SARS-CoV-2 regardless of the epidemiological investigation's findings as previously reported, and any positive case, regardless of symptoms or viral load (as assessed by N-gene Ct-value) was isolated. At the same time, an extensive investigation was conducted to trace specific gaps in PPE usage and in order to define specific types of contacts, which might attribute to the occurrence of the outbreak. The IPCU team visited the laundromat facility several times, to observe the workflow and fill gaps in the epidemiological investigation. Following the initial findings, several measures were taken: public showers use was limited to one person at a time; dividers were constructed along with the sorting table in order to separate the workers along with the station and ensure proper distance, and the mandatory usage of face shields and N-95 masks was regulated. Even though commuting, eating and smoking together was formally banned since the beginning of the pandemic, we re-emphasised that these high-risk behaviours should be avoided and regulated.

## Discussion

Here we present a nosocomial outbreak of SARS-CoV-2 among non-clinical HCW, integrating an epidemiologic investigation with next-generation genome sequencing of the SARS-CoV-2 isolates. The integration of the two allowed us to determine the probable routes of transmission. We were able to demonstrate that within a nosocomial SARS-CoV-2 outbreak among laundry HCW, which coincided with the second COVID-19 surge in Israel, there were three different viral strains, with three suspected index cases in a single department. For each of the index cases, there was a putative community source of infection. While we initially suspected that the source of infection could be the intense handling of dirty laundry from the COVID-19-dedicated units, the integrated investigation suggested person to person transmission routes.

Previous reports on SARS and MERS have demonstrated potential transmission from contaminated cotton gowns to laundromat HCW [14, 15]. Currently, the role of environmental transmission of SARS-CoV-2 has been largely debated [2, 4, 5, 16], and the main hypothesis is that SARS-CoV-2 is mostly transmitted from person to person via droplets [2] or less commonly via aerosol [17]. Attempts to detect the viable virus by infectivity assays from environmental samples had mostly fallen short [2, 6, 18]. Our study suggested three different index cases for three chains of transmission, making an environmental source less probable.

We found that the most common source of transmission is direct person-to-person contact among pre-symptomatic or asymptomatic HCW. Transmission events mostly occurred due to improper PPE usage during shared working and social gatherings. Specific high-risk situations in which most transmission events occurred, included shared commute, simultaneous use of communal showers and dining or drinking together. Direct observations by the IPCU team revealed that most workers used surgical masks instead of N-95, did not use face shields, and were unable to maintain a proper distance from one another.

The environmental setting of the laundromat facility is a high-risk setting which requires the usage of masks [19–21]. The temperature in the laundromat premises can be extremely hot due to the machines' heat emission and the warm Israeli summer weather, when the outbreak occurred. It has been demonstrated that wearing masks while working may increase facial skin temperature at a level that may induce thermal discomfort [22]. Additionally, people tend to touch their masks as many as 8–25 times per hour [22]. We suspect that the combination of the environmental setting, the physical labour and the heating effect of the masks, made it hard to comply with protection measures, which contributed to the outbreak's occurrence.

A significant and frequently overlooked route of transmission in the current outbreak is the shared commute. Viral linage analyses confirmed commute in private vehicles to be the probable route of transmission in two clusters. A recent study describes how vehicles' cabin microclimates affect airborne infection transmission and the recommended open\closed window pattern [23]. The patients in our cohort who commuted together and belong to the same cluster did not use proper PPE while commuting and left the vehicles' windows closed during the ride.

Another finding was the usage of communal showers as a potential risk for COVID-19 transmission. In our cohort, three pairs of patients used the public showers of the laundromat facility at the same time, in proximity, during symptom onset and repeatedly thereafter. Only two pairs are from the same clusters. While this might suggest that showering together harbours a risk for infection transmission, the four HCW with a similar lineage had also worked together on several occasions. Furthermore, temperature and humidity were shown to have an impact on viral transmission: the higher the temperature and the humidity, the lower the infectivity [24]. Nevertheless, a possible SARS-CoV-2 transmission in a public bath centre has been reported [25].

In conclusion, the combination of molecular and epidemiological investigation made it possible to stratify and indicate which types of contacts are high-risk for SARS-CoV-2 transmission. We report that a shared working schedule and shared commute in a closed vehicle, with reduced compliance to PPE usage due to high temperature and humidity, serve as major risk factors for infection transmission among non-clinical HCW, while the chance of transmission from using communal showers may be lower. We are unable to make a single definite statement about the potential of infection transmission from dirty laundry, yet, the results reported here do not support this route of transmission. Further data should be collected on the matter to clarify whether such transmission is likely.

This study has several limitations. First, we were unable to sequence samples from all 14 infected cohort cases since some HCW were tested in the community and their isolates were not available. Second, we did not have samples from contaminated textiles to rule out the dirty laundry as a source of transmission.

Third, since the epidemiologic investigation is based largely on interviews and questionnaires, it is prone to recall bias [26] and the potential mandatory 14-days quarantine which is imposed on exposed HCW, potentially led to distorted answers and information bias. Therefore, we repeated some of the interviews, cross-referenced certain answers and interviewed the HCW's supervisors and assessed their work schedule.

## Conclusion

In summary, we report an extensive epidemiological and molecular investigation of a SARS – CoV-2 outbreak among laundry workers, elucidating the likely routes of transmission in a COVID-19 outbreak. We demonstrate that shared commute, eating or drinking together and possibly using communal showers simultaneously, are high-risk situations for infection transmission. Proper PPE usage and social distancing are not sufficient to prevent nosocomial outbreaks in such settings..

The authors declare no conflict of interest. This study was self-funded by the SMC. The data from the epidemiological investigations are not available to the public due to patients doctors confidentiality.

## Data Availability

The data that support the findings of this study is partially available; Sequences are available in the GISAID database, under the following accession numbers: EPI_ISL_3000158, EPI_ISL_3002932, EPI_ISL_3000159, EPI_ISL_3003644, EPI_ISL_3003444, EPI_ISL_3003268, EPI_ISL_3003109, EPI_ISL_3002726, EPI_ISL_3002494, EPI_ISL_3002161, EPI_ISL_3000156. Phylogenetic trees were made by MEGAX. Bioinformatics and phylogenetic tree were analysed using R and Bioconductor, with a description of the analysis process at doi: 10.3390/v12080854 [10]. The answers to the epidemiological investigation questioners are protected under patient-doctor confidentiality, thus cannot be uploaded to a public database. We can provide de-identified data upon request.
